# SENOTHERAPEUTICS, AGING, AND AGE-RELATED DISEASES

**DOI:** 10.1093/geroni/igae098.1474

**Published:** 2024-12-31

**Authors:** Dean Kellogg

**Affiliations:** The University of Texas Health Science Center San Antonio, San Antonio, Texas, United States

## Abstract

Increased translational clinical trials are addressing the utility of senotherapeutic agents as interventions for aging in general and for age-related diseases. Numerous pre-clinical trials have provided a good rationale for human clinical trials which are being increasingly proposed. At present a number of senolytic and senomorphic drugs appear to be safe and tolerable by older persons, hence new human clinical trials directed at efficacy in ameliorating age-related diseases and aging in general are now coming to the fore. This talk will review:1) what are these agents? 2) what are their mechanism(s) of action? and 3) what efficacy have they demonstrated?

